# Quadriceps Tendon Rupture in an Emergency Department Patient with Left Knee Pain

**DOI:** 10.3390/diagnostics16172695

**Published:** 2026-08-24

**Authors:** Andrew G. Theophanous, Rebecca G. Theophanous

**Affiliations:** 1 University of Toledo College of Medicine, 3000 Arlington Ave, Toledo, OH 43614, USA; andrew.theophanous@rockets.utoledo.edu; 2 Department of Emergency Medicine, Duke University School of Medicine, 2301 Erwin Rd, Durham, NC 27705, USA; 3 Durham Veterans Affairs Healthcare System, Durham, NC 27710, USA

**Keywords:** tendon rupture, point-of-care ultrasound, diagnostic reasoning

## Abstract

Point-of-care ultrasound (POCUS) is a dynamic imaging modality that can diagnose musculoskeletal and tendon injuries to facilitate appropriate orthopedic care and improve patient outcomes. This manuscript illustrates the value of dynamic POCUS to distinguish between complete and partial tendon injuries at the patient’s bedside, which is important for management and disposition planning in emergency department (ED) or clinic patients with acute trauma. In this case, a healthy 34-year-old male presented with acute left knee pain and swelling after a fall. He had a deformity superior to his patella and could not actively extend his left knee. Left knee X-ray was negative for fracture. POCUS demonstrated a disrupted left distal quadriceps tendon with anechoic fluid, tendon enlargement, and proximal tendon retraction. Importantly, a dynamic POCUS video cine loop revealed no contiguous tendon mobility with passive extension of the left knee (when movement is performed by the physician), confirming that this was a complete and not partial tendon tear. The patient was discharged in an extension knee brace. Outpatient magnetic resonance imaging confirmed the complete left quadriceps tendon rupture, and he underwent orthopedic surgery with good outcomes. Thus, early dynamic musculoskeletal POCUS confirmed a complete tendon injury, guiding expedited surgical treatment to prevent permanent damage and disability.

**Figure 1:**
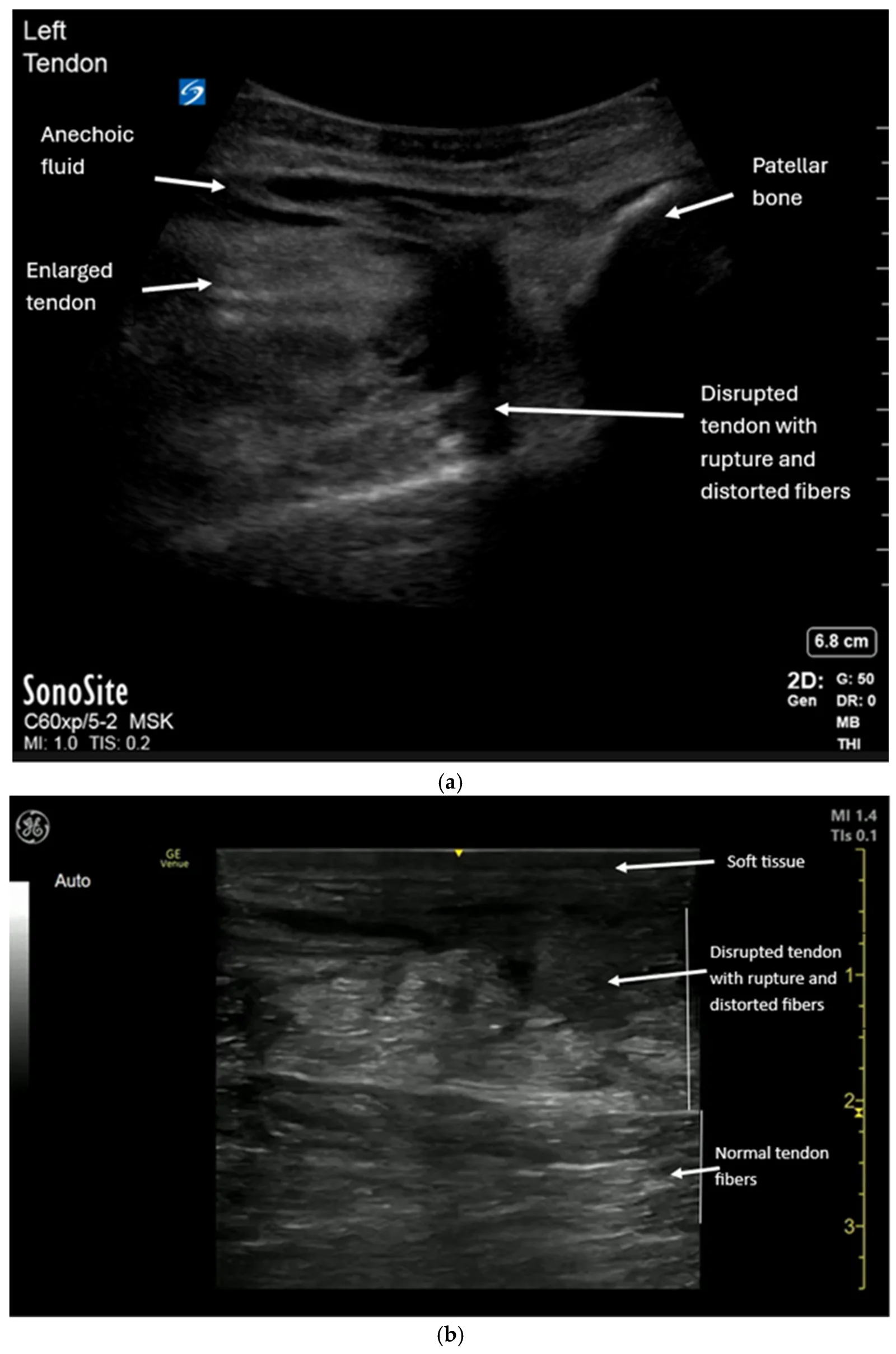
Dynamic point-of-care ultrasound (POCUS) can be performed at the patient’s bedside, provides rapid diagnosis, and helps guide clinical management for musculoskeletal injuries, especially in emergency department (ED) patients with recent trauma. (**a**) demonstrates a complete quadriceps tendon rupture with an enlarged tendon and tendon retraction away from the patellar bone, which cannot be easily seen on radiography or computed tomography [1]. Background: Quadriceps tendon rupture is a lower limb injury that involves suprapatellar pain, loss of knee extension, and a difficult rehabilitation course [1,2,3]. Tendon rupture occurs during eccentric contraction of the quadriceps with knee flexion against an active quadriceps muscle or when a person falls backwards with a planted foot [3,4]. Incidence is 1.37 of 100,000 cases and more frequent in patients over 40 years old [3,4]. Tendon rupture can be spontaneous, particularly in patients with predisposing risk factors such as prior tendon injury, underlying tendinopathy, or comorbidities such as rheumatologic disease, chronic kidney disease, or diabetes mellitus [4,5]. Clinical diagnosis of complete rupture includes distal thigh tenderness, a suprapatellar hematoma, and inability to actively extend the knee. Clinical presentation: These example images are from a healthy 34-year-old male who presented to the ED with acute left knee pain and swelling after landing on his left knee while playing basketball three days prior (**a**). He felt a pop with immediate pain and subsequent swelling. On examination, he had severe tenderness at his left distal thigh/proximal knee with a deformity superior to his patella. His pain was significantly worse with knee movement, and he was unable to stand and bear weight on the leg. Lower extremity strength, sensation, and distal pulses were normal, but he could not extend his left knee. Diagnostic imaging findings: An X-ray of his left knee showed a joint effusion with soft tissue swelling anterior to the patella but no acute fracture. As shown in (**a**), POCUS demonstrated a disrupted left distal quadriceps tendon with anechoic fluid, tendon enlargement near the insertion site, and tendon fiber distortion consistent with a complete tendon rupture (Supplementary Video S1). This is distinct from partial tears, which would show only partial disruption of the tendon, with hyperechoic fibers still attached to the bone distally in the unaffected portion of the tendon (**b**) (Supplementary Video S2). Diagnostic imaging is paramount to confirming tendon rupture diagnosis, and imaging availability, operator expertise, and clinical pathways vary across institutions. Imaging is important for injury confirmation and severity grading to guide management [3]. Radiography may show an inferiorly displaced patella with a patellar length ratio < 0.8, but this finding is not sensitive or specific [4]. Magnetic resonance imaging (MRI) is the gold standard but may be difficult to obtain in emergent or primary clinic settings, whereas performing POCUS can be a facile tool to evaluate for tendon rupture at the patient’s bedside for immediate critical management decisions [3,4,6].

**Figure 2:**
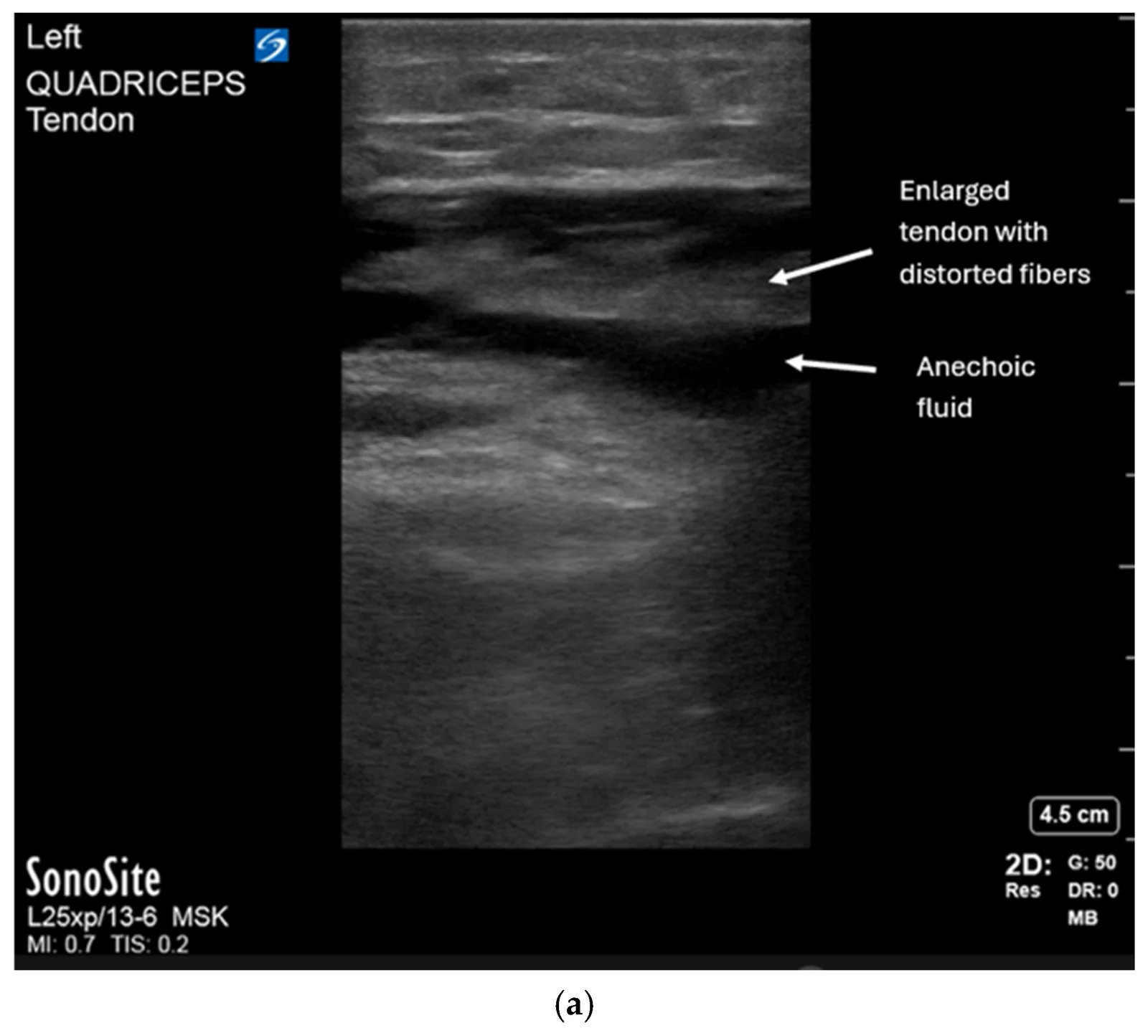

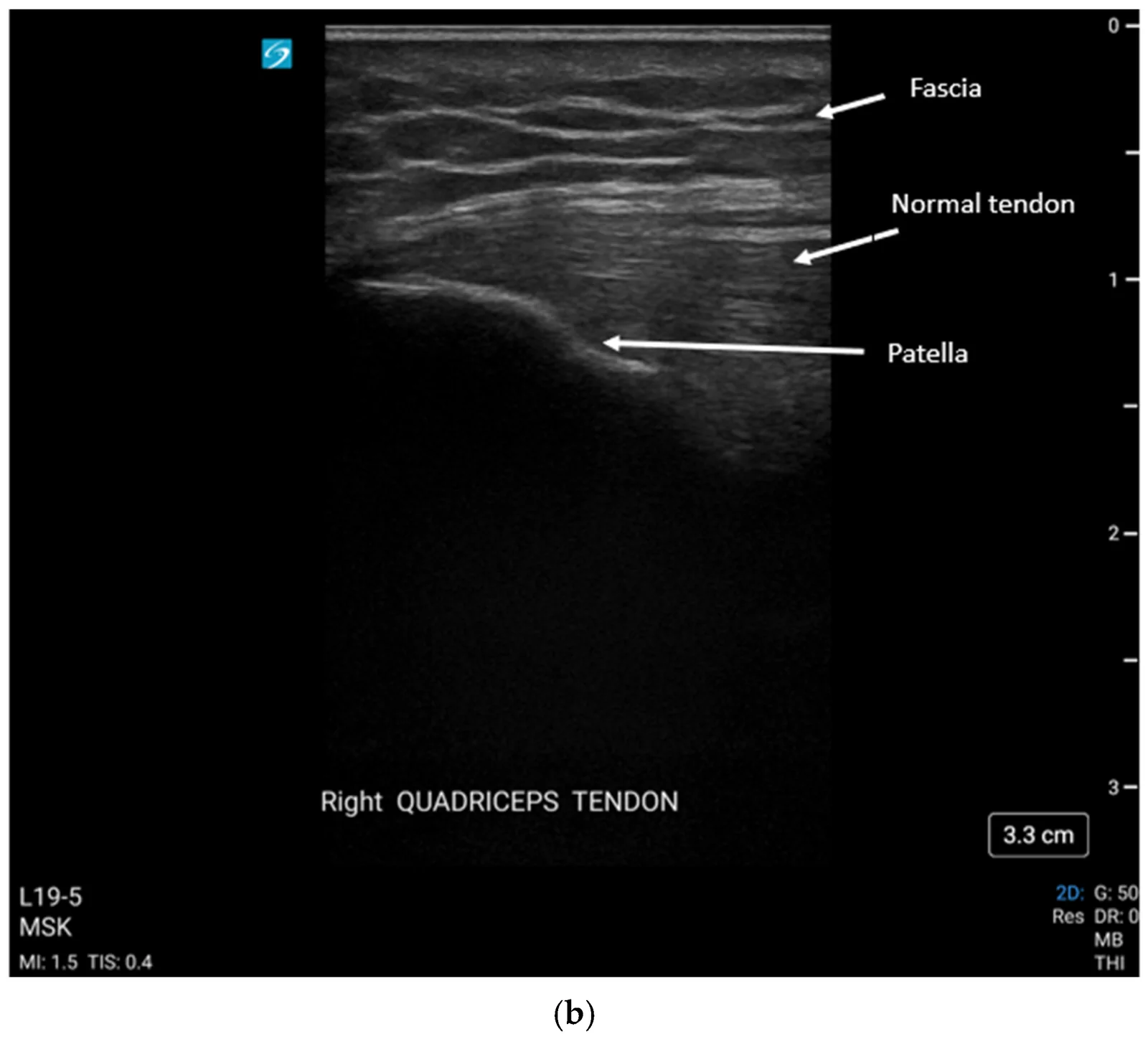
Musculoskeletal ultrasound can clearly visualize tendon injury with comparison of the contralateral side. (**a**) is a transverse point-of-care ultrasound image demonstrating the key diagnostic POCUS findings with acute tendon injury, including an enlarged tendon with distorted fibers and anechoic fluid. (**b**) shows the normal contralateral quadriceps tendon. POCUS technique and interpretation: On POCUS, tendons are more echogenic and densely striated compared to muscle. On longitudinal view, intact tendons have a narrow, densely striped fibrillar appearance in parallel lines. (**b**) is an example still image and Supplementary Video S3 shows the normal contralateral quadriceps tendon. On transverse view, the tendon is round or oval with a punctate interior [6]. As illustrated in this case, it is important to assess for dynamic findings on POCUS using video capture to differentiate complete from partial tendon rupture. Supplementary Video S1 is a dynamic POCUS video showing tendon retraction and lack of mobility during range of motion testing, which is specific for complete rupture [6], whereas a partial tear will still have tendon fibers attached at the bony insertion site with some tendon movement during flexion and extension [6] (Supplementary Video S2). Supplementary Video S4 shows the normal contralateral quadriceps tendon with active range of motion. Expedited diagnosis is important because proper treatment of quadriceps tendon rupture, especially in high-risk patients, is crucial to prevent permanent injury and disability. Management varies depending on the patient and degree of injury. Partial-thickness tears may be treated non-operatively while operative repair is common for full-thickness tears, chronic ruptures, and high-grade partial-thickness tears. In this case the patient was discharged in an extension knee brace, and an outpatient knee MRI confirmed a complete left quadriceps tendon rupture with surrounding fluid, hematoma, and tendon retraction. The patient had urgent orthopedic surgical repair and early functional mobilization with range-of-motion exercises, with good recovery of muscle function within the next few months. Management implications: POCUS integration into clinical pathways can help distinguish between causes of acute left knee injuries presenting with an extension deficit. This includes a displaced meniscus tear or a ligament tear, which are diagnosed with free fluid and distorted structures at the medial or lateral aspect of the knee rather than superiorly with a quadriceps tendon injury [5,6,7]. Other injuries include patellar dislocation (most often with a lateral deformity on exam), patellar tendon rupture (deformity with free fluid and distorted fibers at the inferior knee on POCUS), patellar fracture (discontinuity of the bony contour on POCUS), or an extensor mechanism disruption [5,6,7,8]. Finally, POCUS users should be aware of limitations including its user dependence, which requires extensive training to obtain good quality images, correctly interpret the images, and integrate findings into medical decision making [6]. Potential POCUS diagnostic pitfalls include artifacts such as anisotropy, which can create dark areas within the tendon that can be confused with tears if the probe is not held perpendicular to the skin. Also, ligaments will appear similar to tendons but are thinner, more irregular, and difficult to see [6]. Users should always image the unaffected tendon for comparison to help locate normal structures such as the bone, tendon, muscle body, and soft tissue layers [6]. Follow-up MRI can confirm tendon rupture diagnosis in uncertain cases. In summary, early diagnosis of complete quadriceps tendon rupture using dynamic POCUS in acute care settings is critical to expedite urgent orthopedic surgical treatment for improved functional outcomes (File S1).

## Data Availability

The original contributions presented in this study are included in the article. Further inquiries can be directed to the corresponding author.

## Supplementary Materials

The following supporting information can be downloaded at https://www.mdpi.com/article/10.3390/diagnostics16172695/s1. Video S1: Dynamic longitudinal point-of-care ultrasound findings of complete quadriceps tendon rupture with an enlarged tendon, distorted fibers at the patellar insertion site, and surrounding anechoic fluid; Video S2: dynamic longitudinal point-of-care ultrasound view of a partial Achilles tendon rupture; Video S3: Longitudinal view of a normal quadriceps tendon; Video S4: Video of a normal quadriceps tendon with active range of motion. File S1: CARE checklist: standard reporting guidelines for case reports.

## Abbreviations

The following abbreviations are used in this manuscript:

POCUS	Point-of-care ultrasound
MRI	Magnetic resonance imaging
ED	Emergency department
