# Supplementary material for: Quadriceps Tendon Rupture in an Emergency Department Patient with Left Knee Pain
Source: Diagnostics (Basel). 2026 Aug 24;16(17):2695. doi: 10.3390/diagnostics16172695 (PMC13565189; doi:10.3390/diagnostics16172695)
Supplement: Supplementary file 1 [file diagnostics-16-02695-s001.zip › File S1. CARE Checklist CR Quad rupture Aug26.pdf]

2013 CARE Checklist

1. **Title** – The diagnosis or intervention of primary focus followed by the words "case report". N/A since this is "interesting images" submission
2. **Key Words** – 2 to 5 key words that identify diagnoses or interventions in this case report (including "case report"). Page 1
3. **Abstract** – (structured or unstructured) Page 1, unstructured
   - Introduction – What is unique about this case and what does it add to the scientific literature? This manuscript illustrates the value of dynamic POCUS to distinguish between com-plete and partial tendon injuries at the patient's bedside, which is important for man-agement and disposition planning in emergency department (ED) or clinic patients with acute trauma. Urgent surgical repair must be performed within several days for complete rupture for good patient outcomes.
   - The patient's main concerns and important clinical findings. A healthy 34-year-old male presented with acute left knee pain and swelling after falling while playing basketball three days prior. On examination, he had severe tenderness at his left distal thigh with a deformity superior to his patella. He had intact strength, sensation, and distal pulses in both legs but could not passively extend his left distal leg. Point-of-care ultrasound (POCUS) demonstrated a disrupted left distal quadriceps tendon with anechoic fluid and tendon enlargement.
   - The primary diagnoses, interventions, and outcomes. The patient was discharged in an extension knee brace, with outpatient magnetic resonance imaging confirming a complete left quadriceps tendon rupture with surrounding fluid and tendon retraction. He underwent urgent orthopaedic surgery with a good functional outcome.
   - Conclusion – What are one or more "take-away" lessons from this case report? Early dynamic musculoskeletal POCUS confirmed a complete tendon injury, guiding expedited surgical treatment to prevent permanent damage and disability.
4. **Introduction** – Briefly summarizes why this case is unique and may include medical literature references. Page 2

5. **Patient Information** **Page 2-3, case presentation**
    - De-identified patient specific information. Page 2, Figure 1
    - Primary concerns and symptoms of the patient. Page 2
    - Medical, family, and psychosocial history including relevant genetic information. Page 2
    - Relevant past interventions and their outcomes. Page 2
6. **Clinical Findings** – Describe significant physical examination (PE) and important clinical findings. **Page 2**
7. **Timeline** – Historical and current information from this episode of care organized as a timeline (figure or table). **Page 2-3, figures are of US images**
8. **Diagnostic Assessment** **Page 2-3, Figure 1&2 US images**
    - Diagnostic methods (PE, laboratory testing, imaging, surveys).
    - Diagnostic challenges.
    - Diagnosis (including other diagnoses considered).
    - Prognostic characteristics when applicable.
9. **Therapeutic Intervention** **Page 3**
    - Types of therapeutic intervention (pharmacologic, surgical, preventive).
    - Administration of therapeutic intervention (dosage, strength, duration).
    - Changes in therapeutic interventions with explanations.
10. **Follow-up and Outcomes** **Page 3**
    - Clinician- and patient-assessed outcomes if available.
    - Important follow-up diagnostic and other test results.
    - Intervention adherence and tolerability. (How was this assessed?)
    - Adverse and unanticipated events.
11. **Discussion** **Page 3-4**
    - Strengths and limitations in your approach to this case.
    - Discussion of the relevant medical literature.
    - The rationale for your conclusions.

- The primary “take-away” lessons from this case report (without references) in a one paragraph conclusion.

12. **Patient Perspective** – The patient should share their perspective on the treatment(s) they received. N/A

13. **Informed Consent** – The patient should give informed consent. (Provide if requested.) listed in declarations and title page, informed consent was obtained per Institutional Review Board Guidelines.

The CARE checklist (and writing outline) have been translated into multiple languages. Visit SWIHM to access these translations.
